# Supplementary material for: Differential Binding of ΔFN3 Proteins of Bifidobacterium longum GT15 and Bifidobacterium bifidum 791 to Cytokines Determined by Surface Plasmon Resonance and De Novo Molecular Modeling
Source: Int J Mol Sci. 2025 Oct 30;26(21):10560. doi: 10.3390/ijms262110560 (PMC12607495; doi:10.3390/ijms262110560)
Supplement: Supplementary file 1 [file ijms-26-10560-s001.zip › ijms-3912627. Supplementary materials corrected version.pdf]

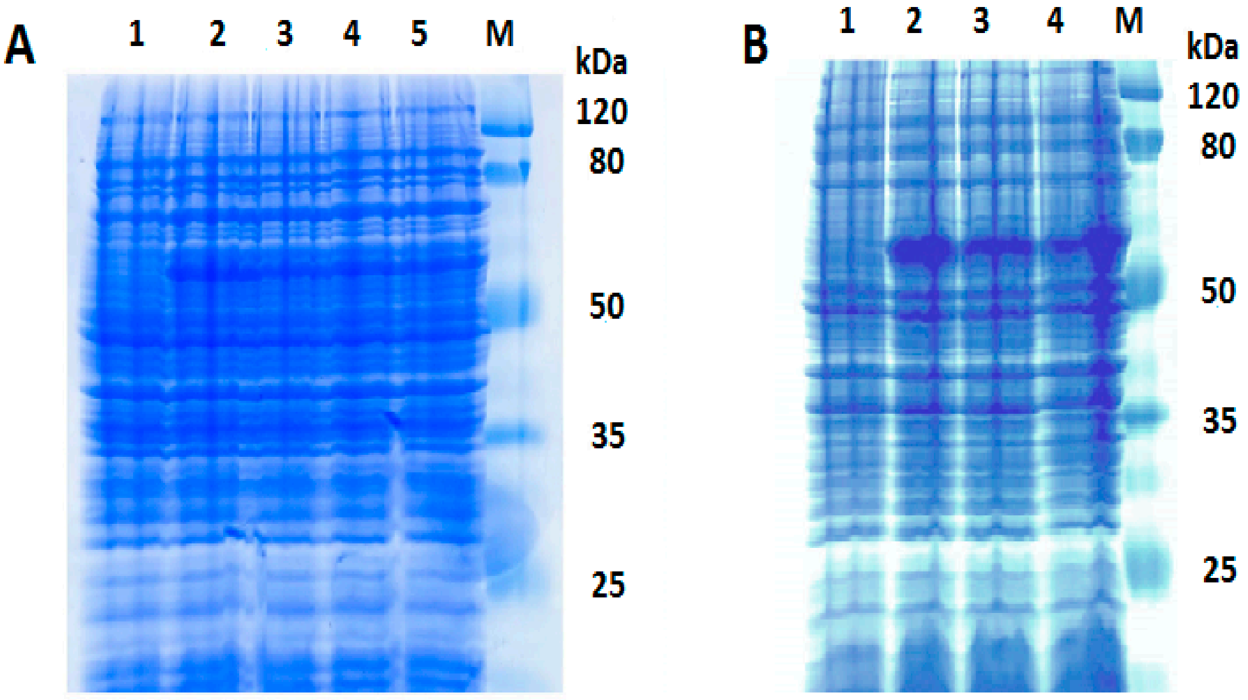

Figure S1. Expression of genes encoding proteins ΔFN3.2 *B. angulatum* GT102 and ΔFN3.3 *B. bifidum* 791.

A – Electropherogram of the soluble protein fraction of ΔFN3.2 protein in *E coli* BL21(DE3) containing plasmids: 1 - pET16b; 2-5 - pET16b:*Δfn3.2*; M - protein molecular weight marker #26612 (Thermo Scientific, Lithuania).

B – Electropherogram of the soluble protein fraction of ΔFN3.3 protein in *E coli* BL21(DE3) containing plasmids: 1 - pET16b; 2-4 - pET16b:*Δfn3.3*; M - protein molecular weight marker #26612 (Thermo Scientific, Lithuania).

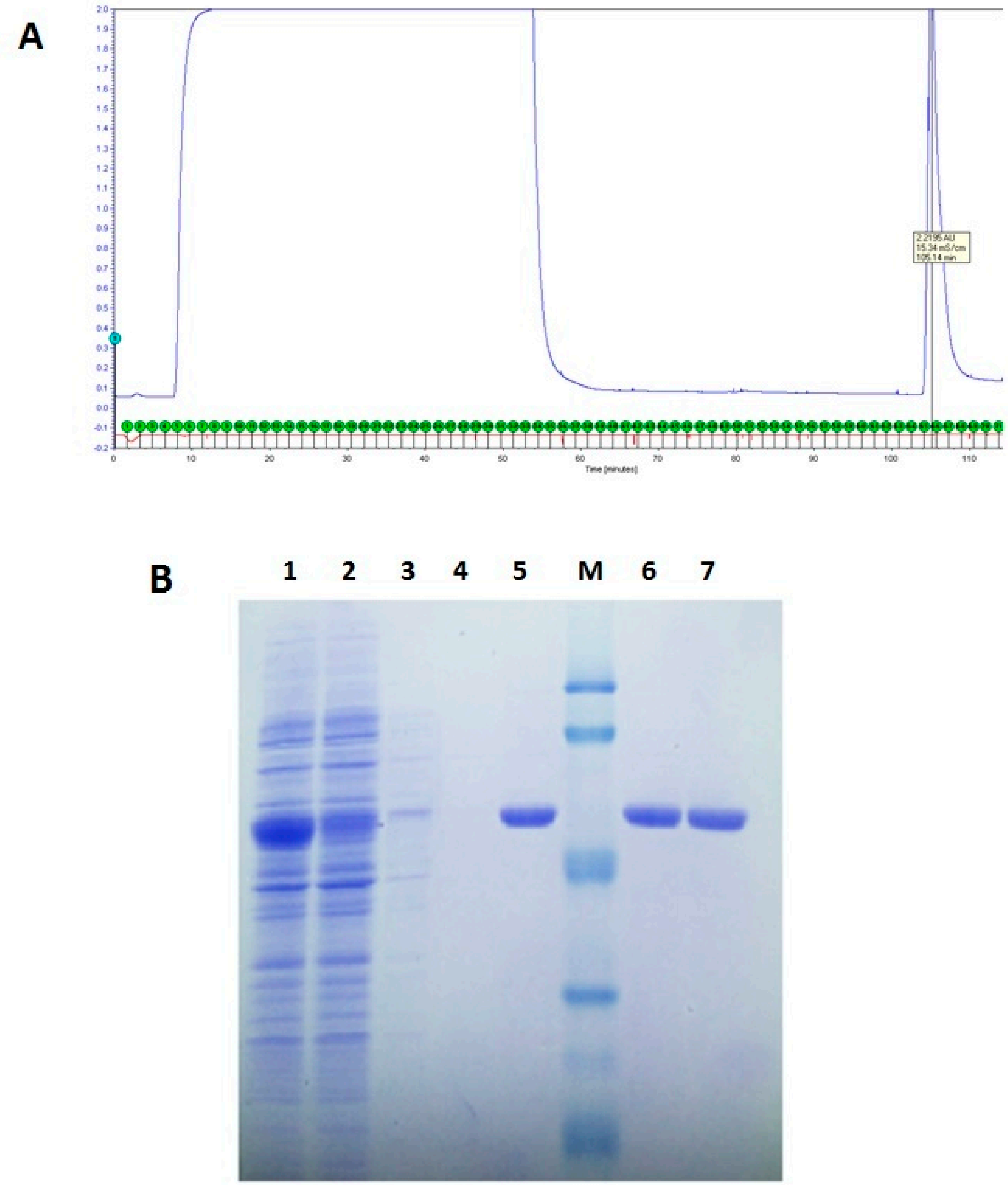

Figure S2. Isolation and purification of recombinant ΔFN3.3 protein.

A - chromatogram of protein isolation and purification.

B - electrophoresis of protein isolation and purification:

1 - lysate; 2 - flow-through; 3 - wash 1; 4 - wash 2; 5-7 – eluates; M - protein molecular weight marker #26612 (Thermo Scientific, Lithuania).

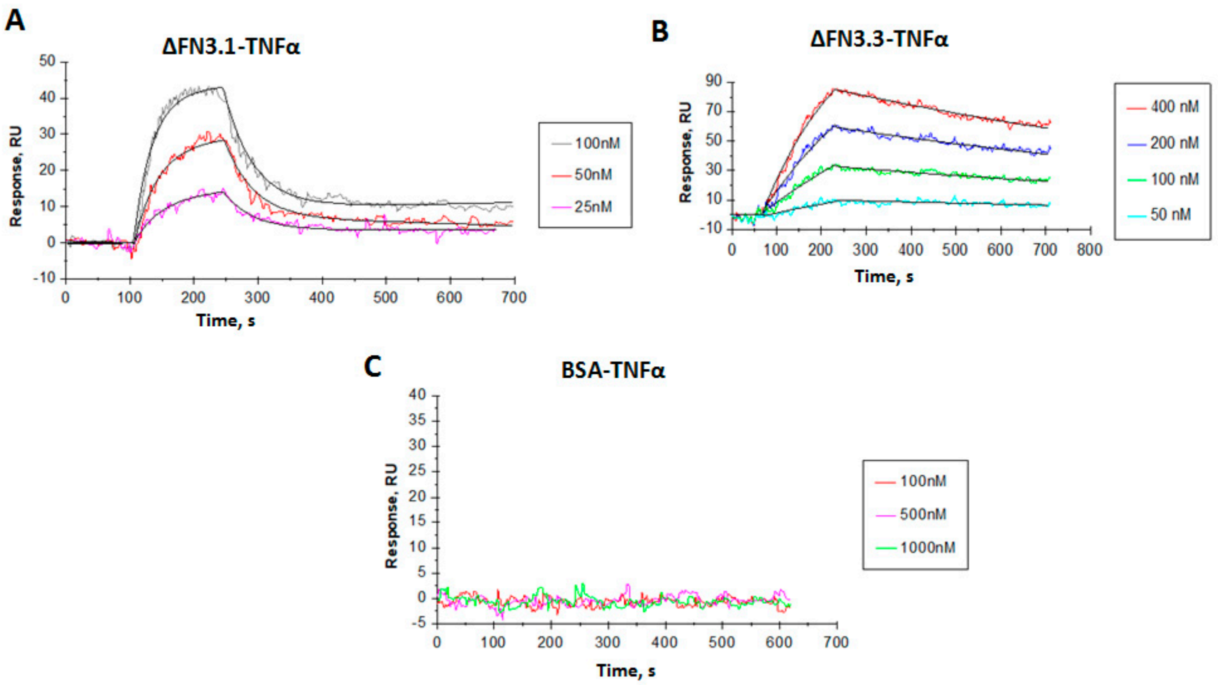

Figure S3. Sensorograms of interaction of TNF-*α* with ΔFN3.1 *B. longum* (A), ΔFN3.3 *B. bifidum* 791 (B) and albumin BSA (C). Black lines correspond to theoretical curves, colored lines to experimental ones.

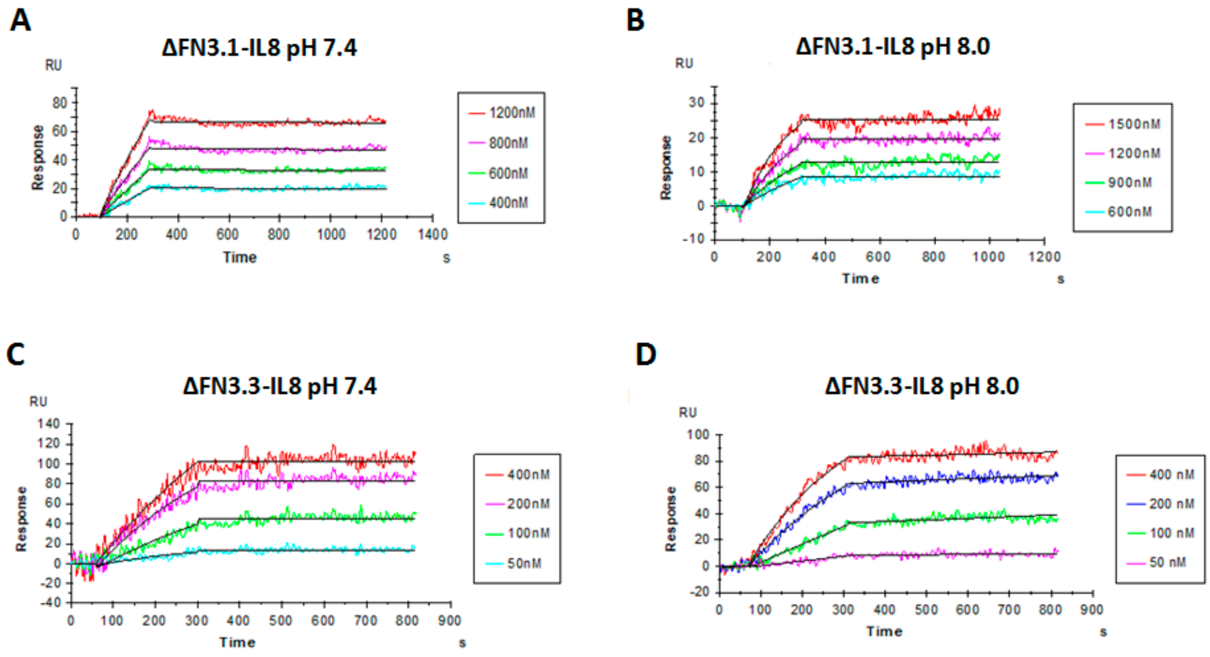

Figure S4. Sensorograms of interaction of ΔFN3.1 *B. longum* GT15 (A,B) and ΔFN3.3 *B. bifidum* 791 (C,D) with IL-8. Black lines correspond to theoretical curves, colored lines to experimental ones.

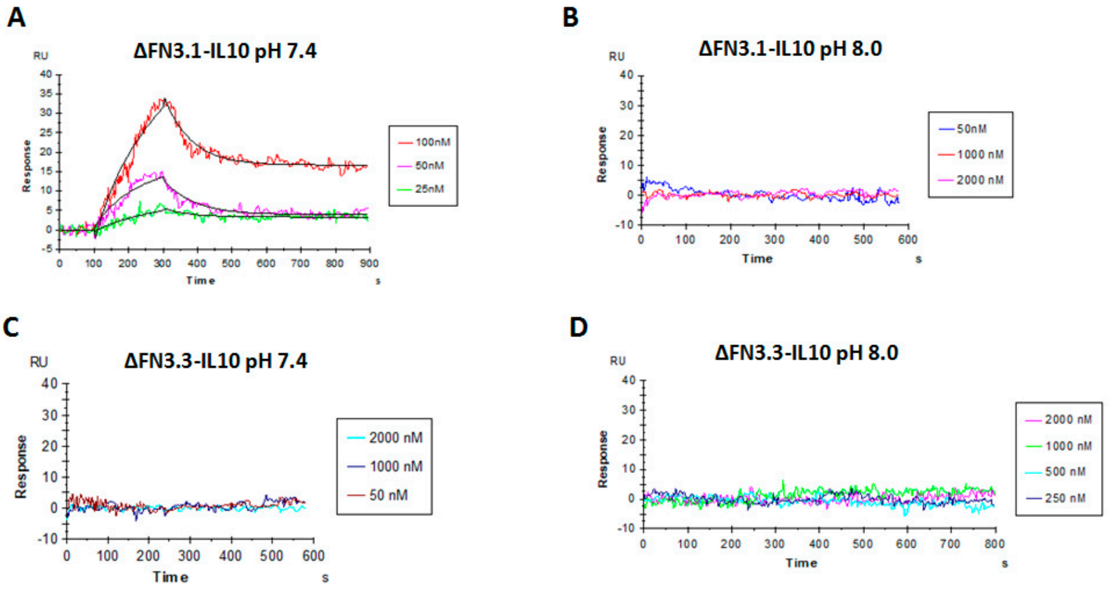

Figure S5. Sensorograms of interaction of ΔFN3.1 *B. longum* GT15 (A,B) and ΔFN3.3 *B. bifidum* 791 (C,D) with IL-10. Black lines correspond to theoretical curves, colored lines to experimental ones.

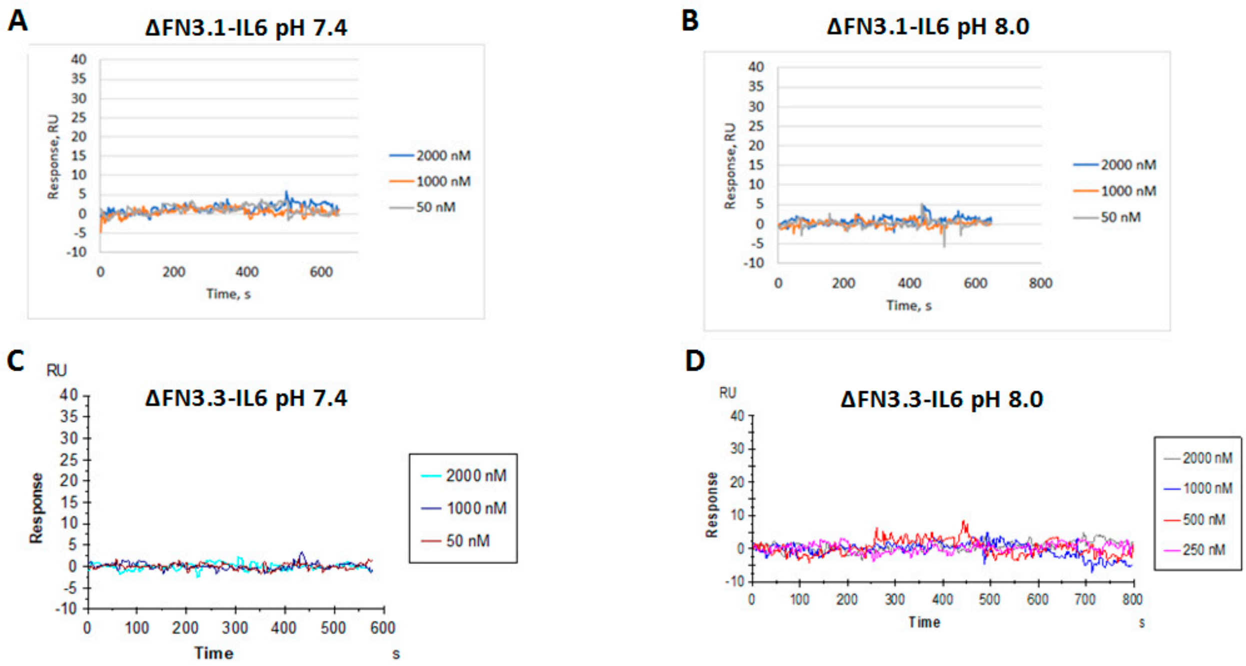

Figure S6. Sensorograms of the interaction of ΔFN3.1 *B. longum* GT15 (A, B) and ΔFN3.3 *B. bifidum* 791 (C,D) with IL-6. Black lines correspond to theoretical curves, colored lines to experimental ones.

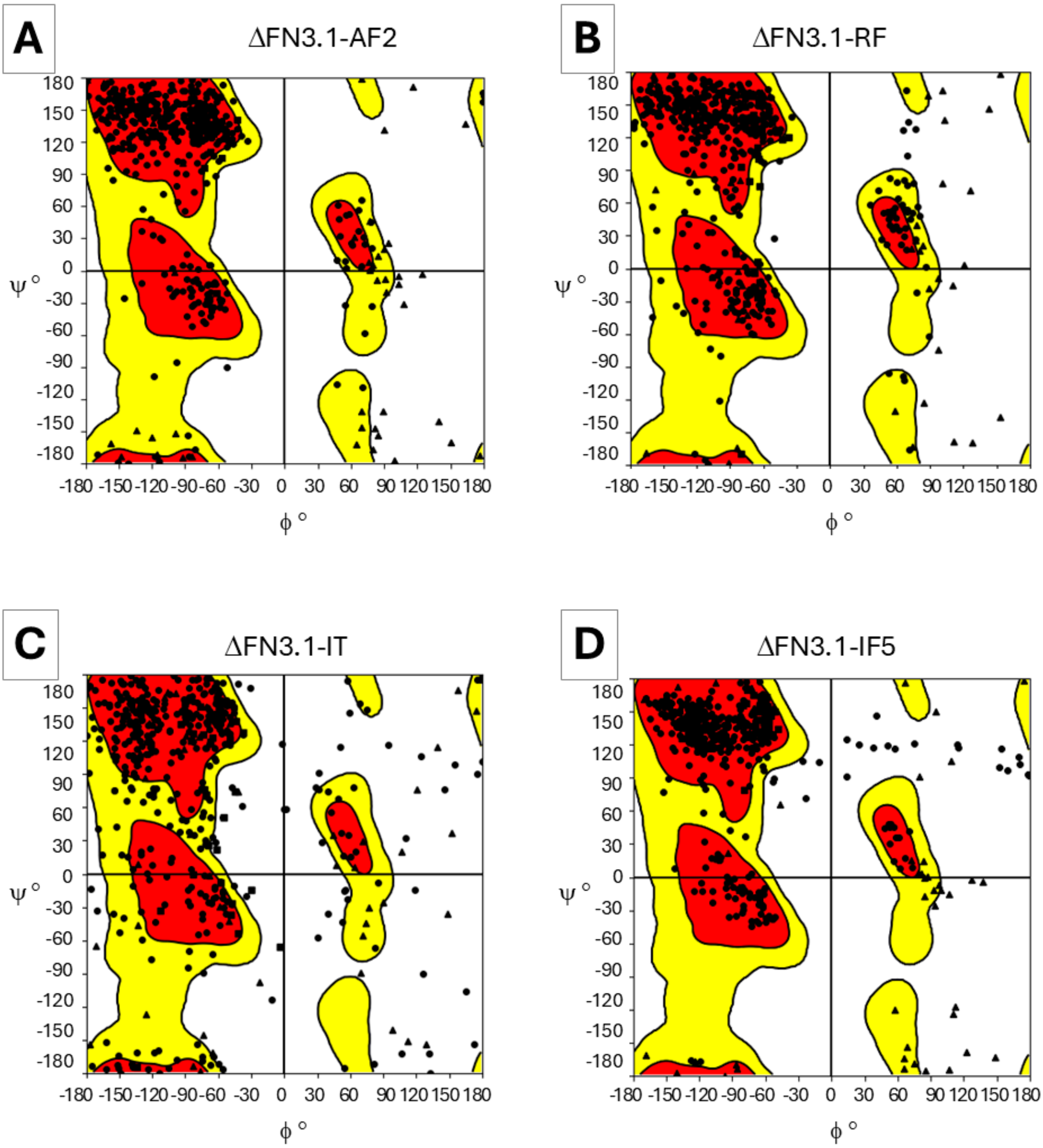

Figure S7. Ramachandran maps for ΔFN3.1 models whose tertiary structure is predicted using various methodologies: AF2, AlphaFold2 (A); RF, RoseTTAFold (B); IT, I-TASSER (C); IF, IntFOLD5 (D).

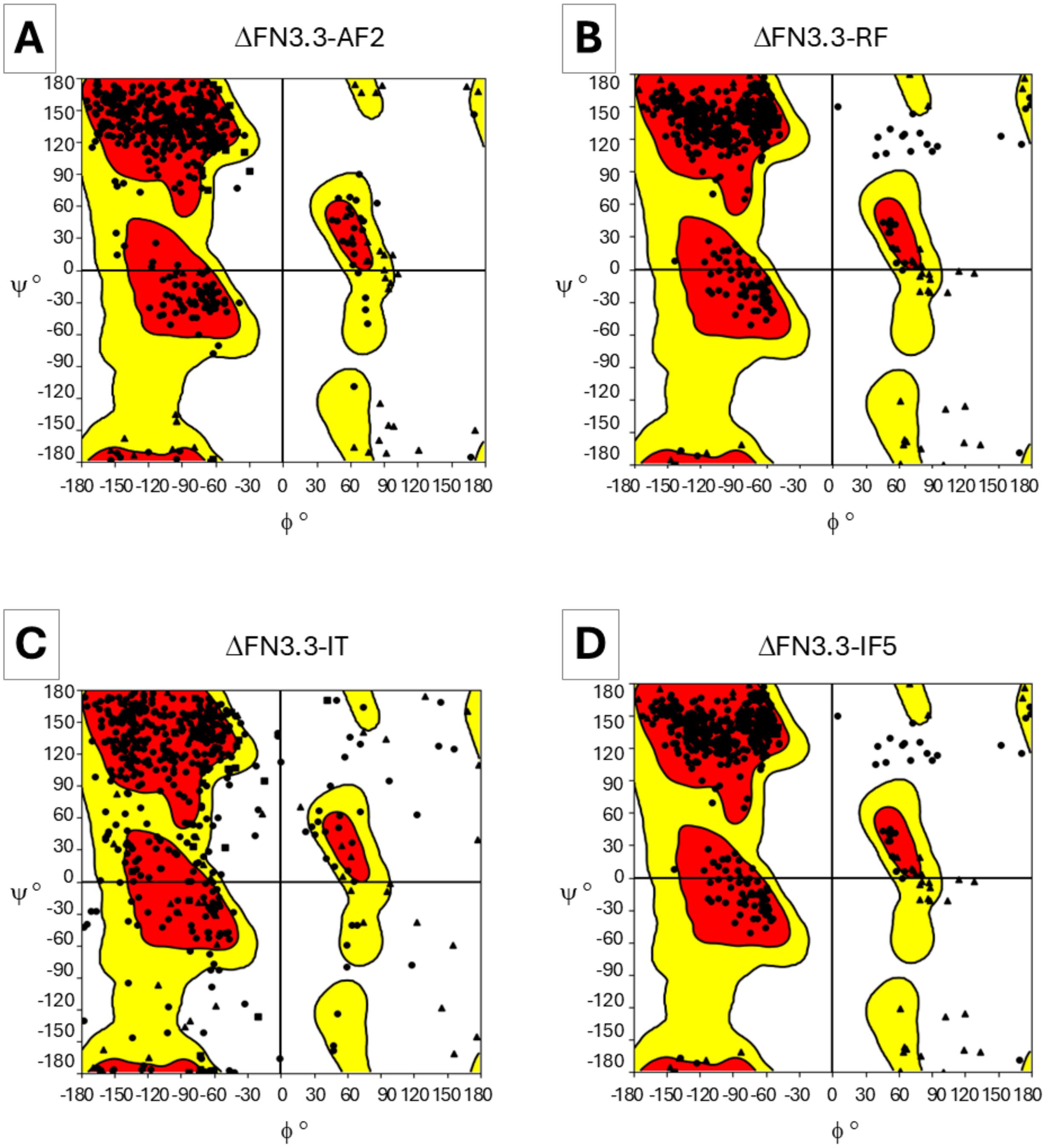

Figure S8. Ramachandran maps for ΔFN3.3 models whose tertiary structure is predicted by various methodologies: AF2, AlphaFold2 (A); RF, RoseTTAFold (B); IT, I-TASSER (C); IF, IntFOLD5 (D).

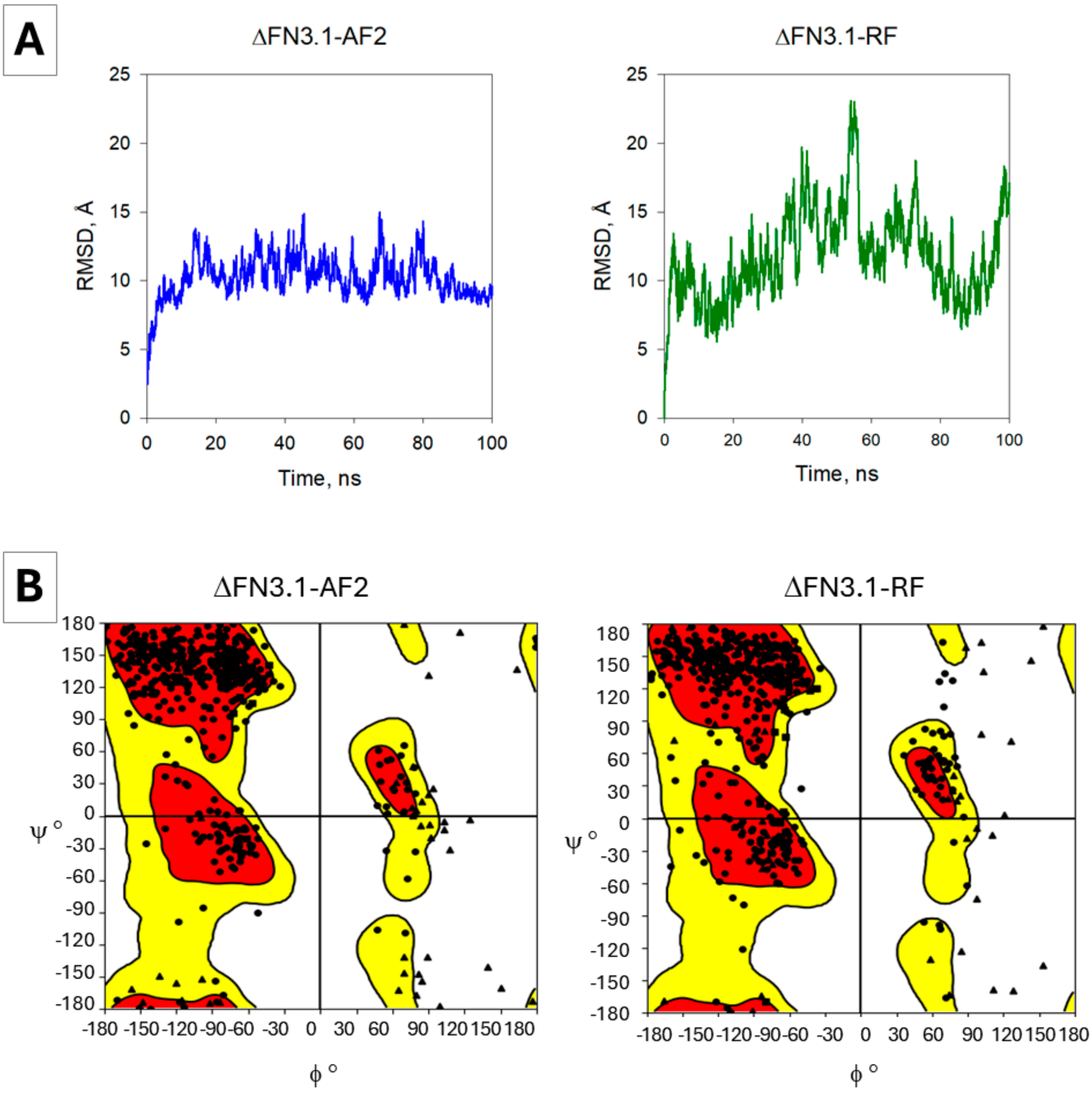

Figure S9. Results of MD simulations of ΔFN3.1 structures predicted by AlphaFold2 (AF2) and RoseTTAFold (RF): RMSD is used to measure average change in displacement of a selection of atoms for a particular frame with respect to a reference frame. RMSD was calculated for all frames in the trajectory (A), Ramachandran maps for representative MD simulation frames (B).

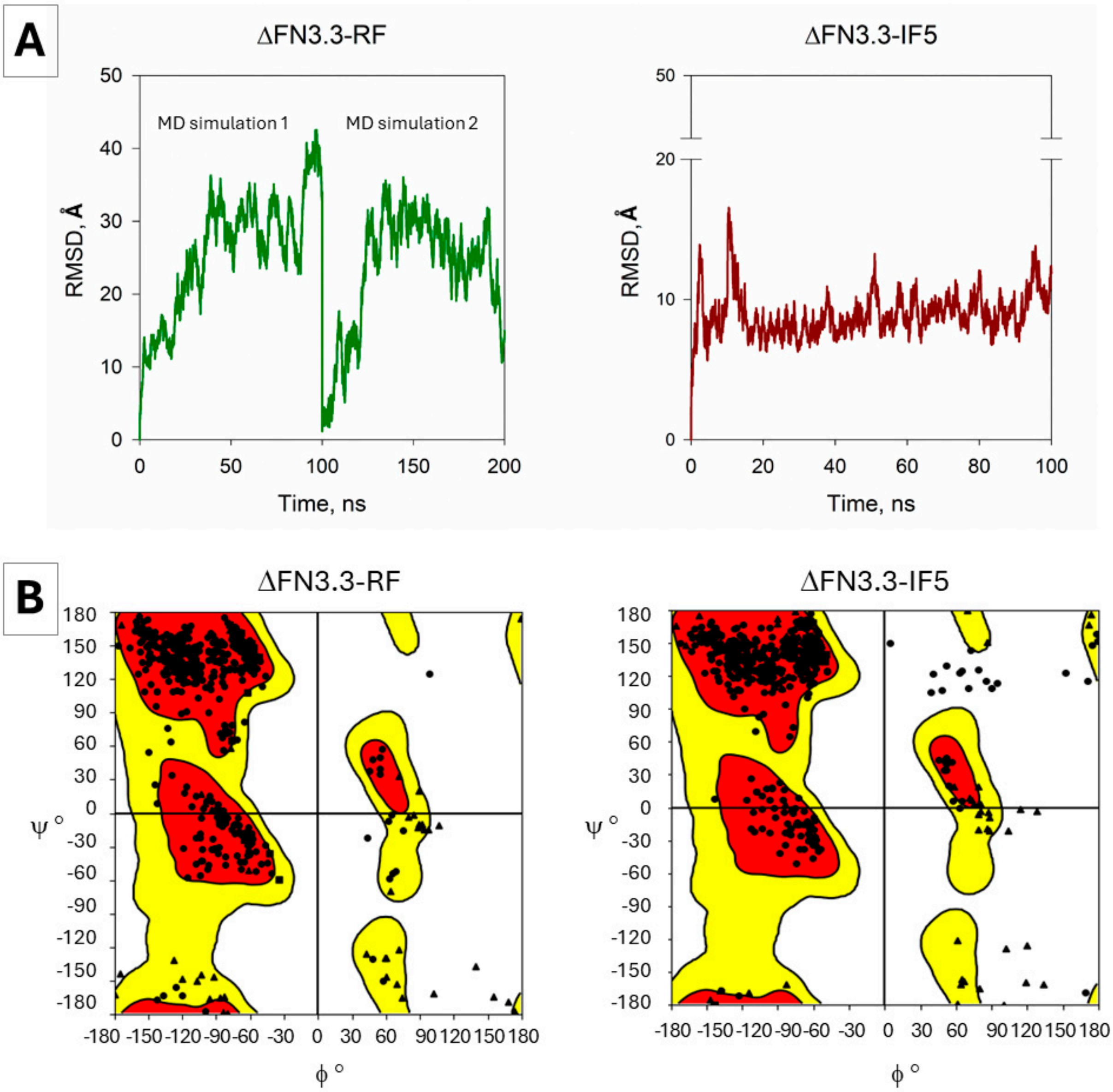

Figure S10. Results of MD simulations of ΔFN3.3 structures predicted by RoseTTAFold (RF) and IntFOLD5 (IF5): RMSD is used to measure average change in displacement of a selection of atoms for a particular frame with respect to a reference frame (calculated for all frames in the trajectory) (A), Ramachandran maps for representative MD simulation frames (B).

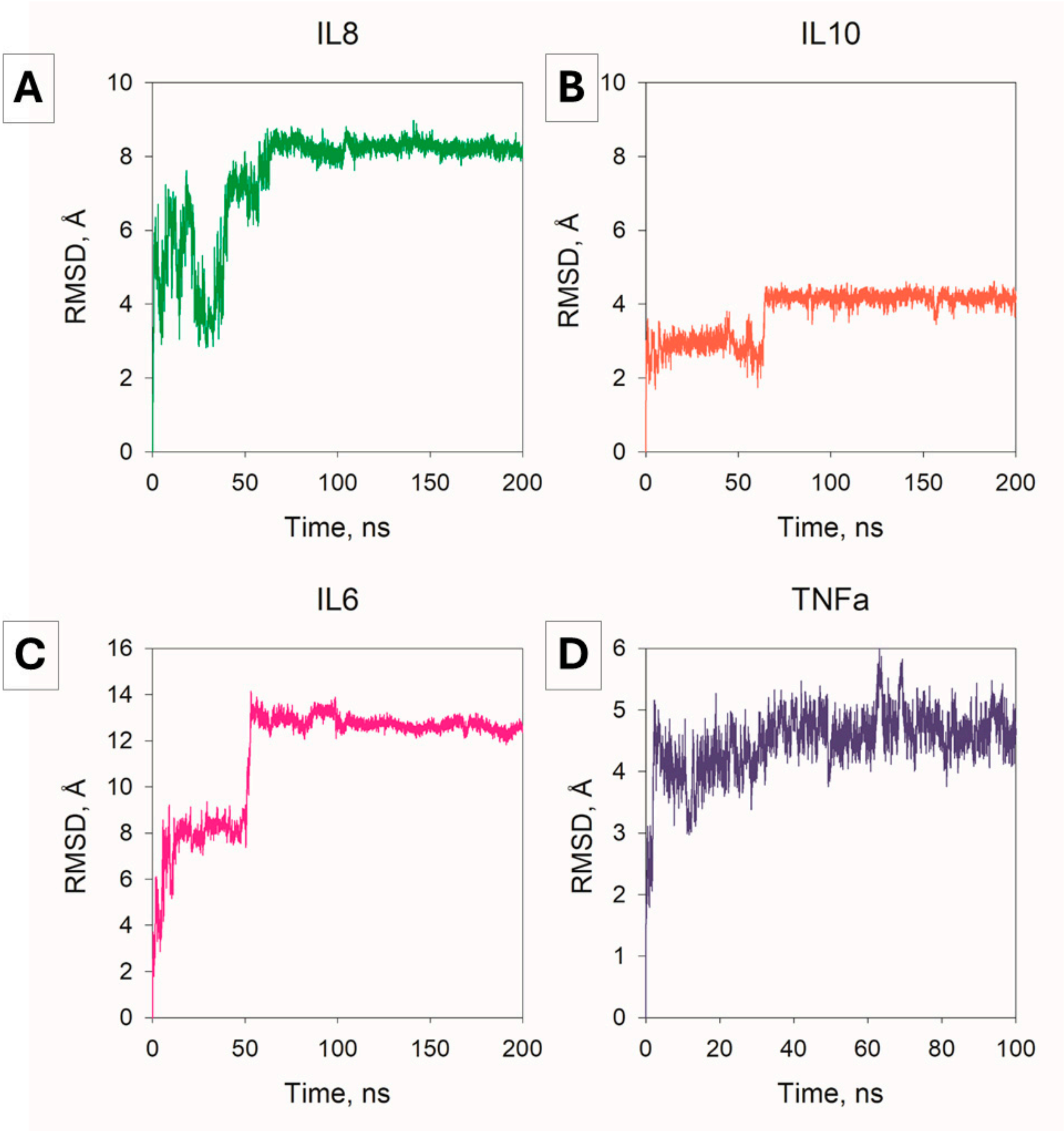

Figure S11. MD simulations of TNF-α and interleukin structures predicted by AlphaFold2: RMSD is used to measure average change in displacement of a selection of atoms for a particular frame with respect to a reference frame calculated for all frames in the trajectory) (A-D).

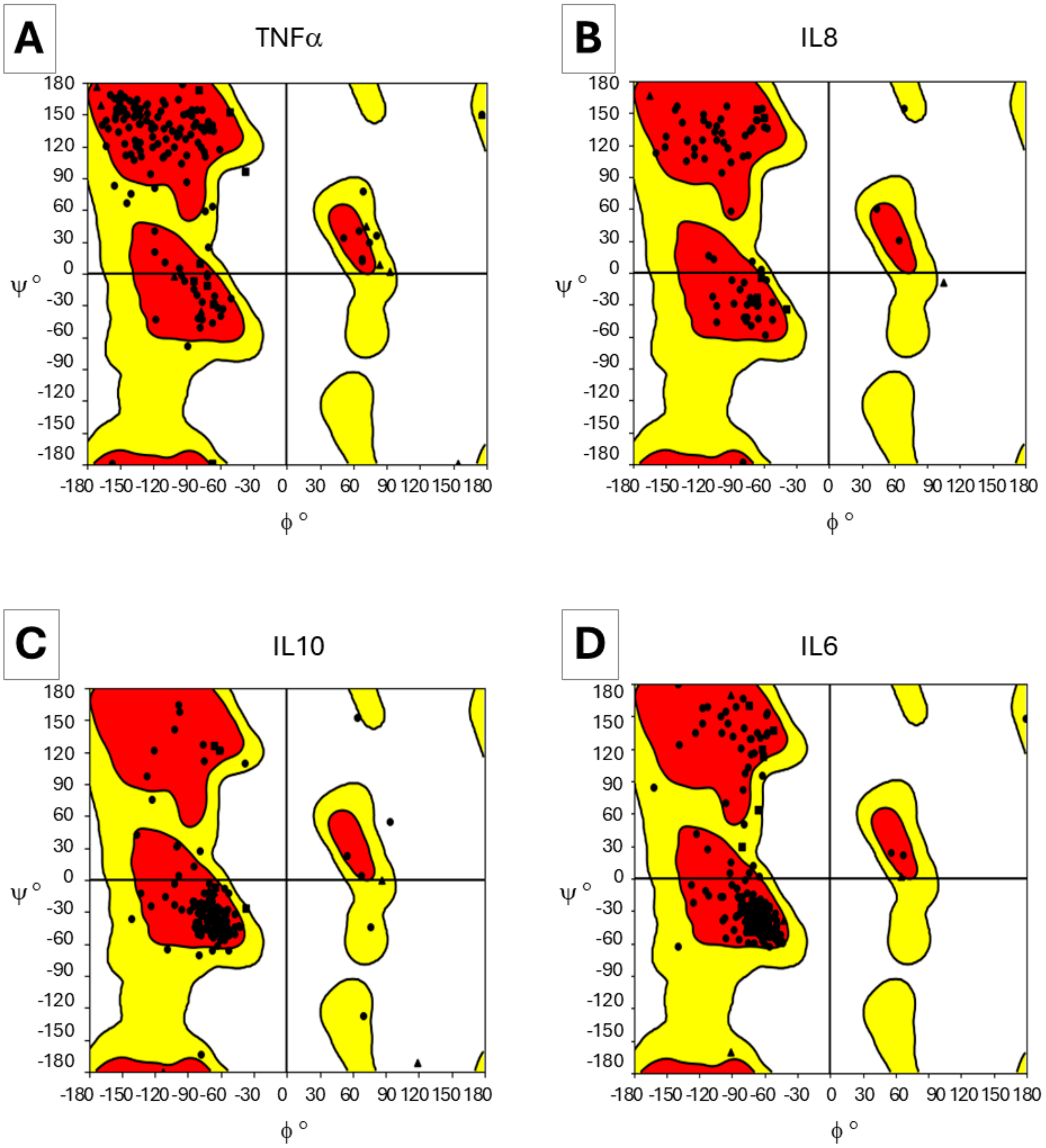

Figure S12. MD simulations of TNF-α and interleukins: Ramachandran maps for representative MD simulation frames (A-D).

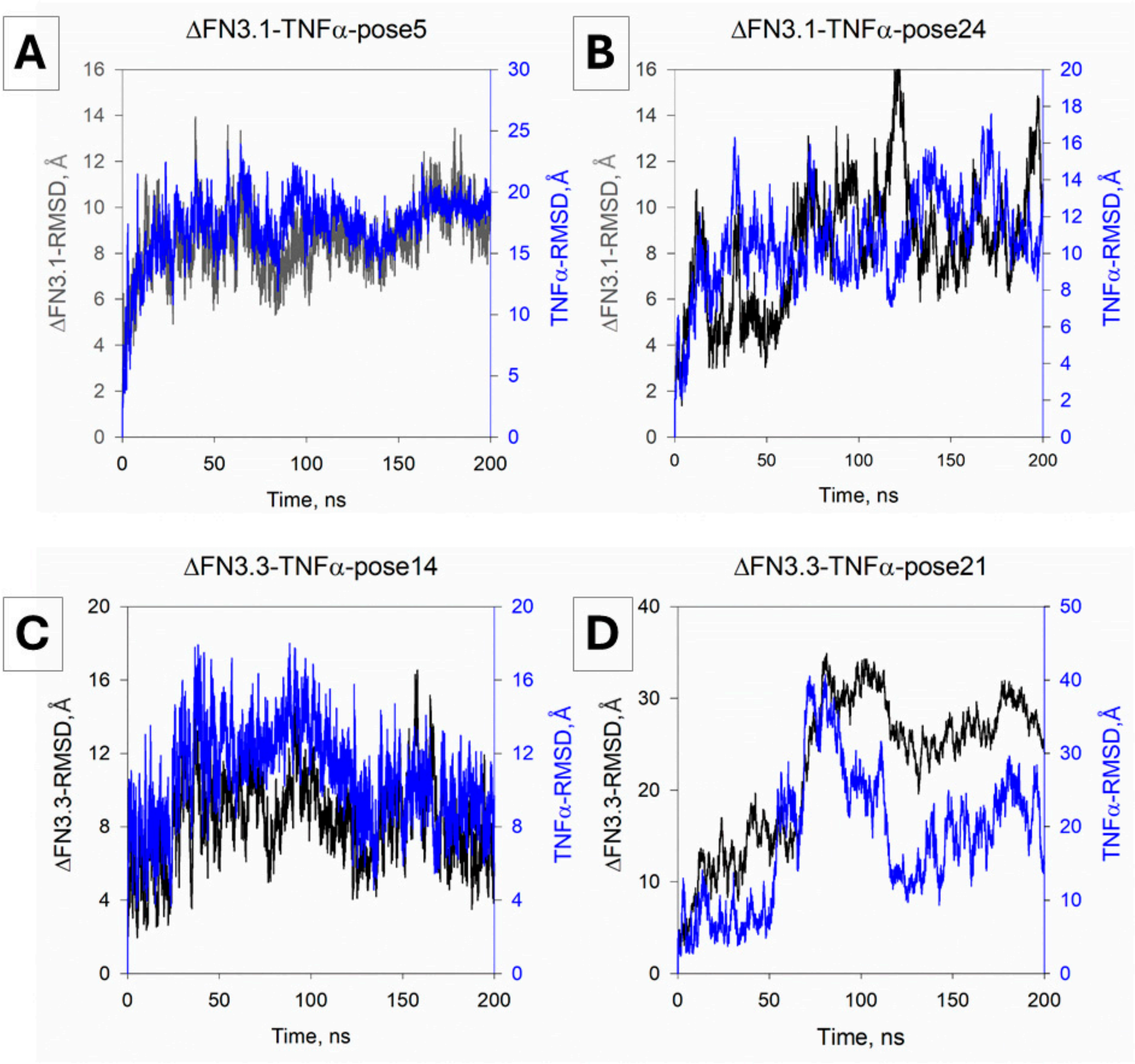

Figure S13. MD simulations of complexes TNF-α-ΔFN3: RMSD is used to measure average change in displacement of a selection of atoms for a particular frame with respect to a reference frame (A-D).

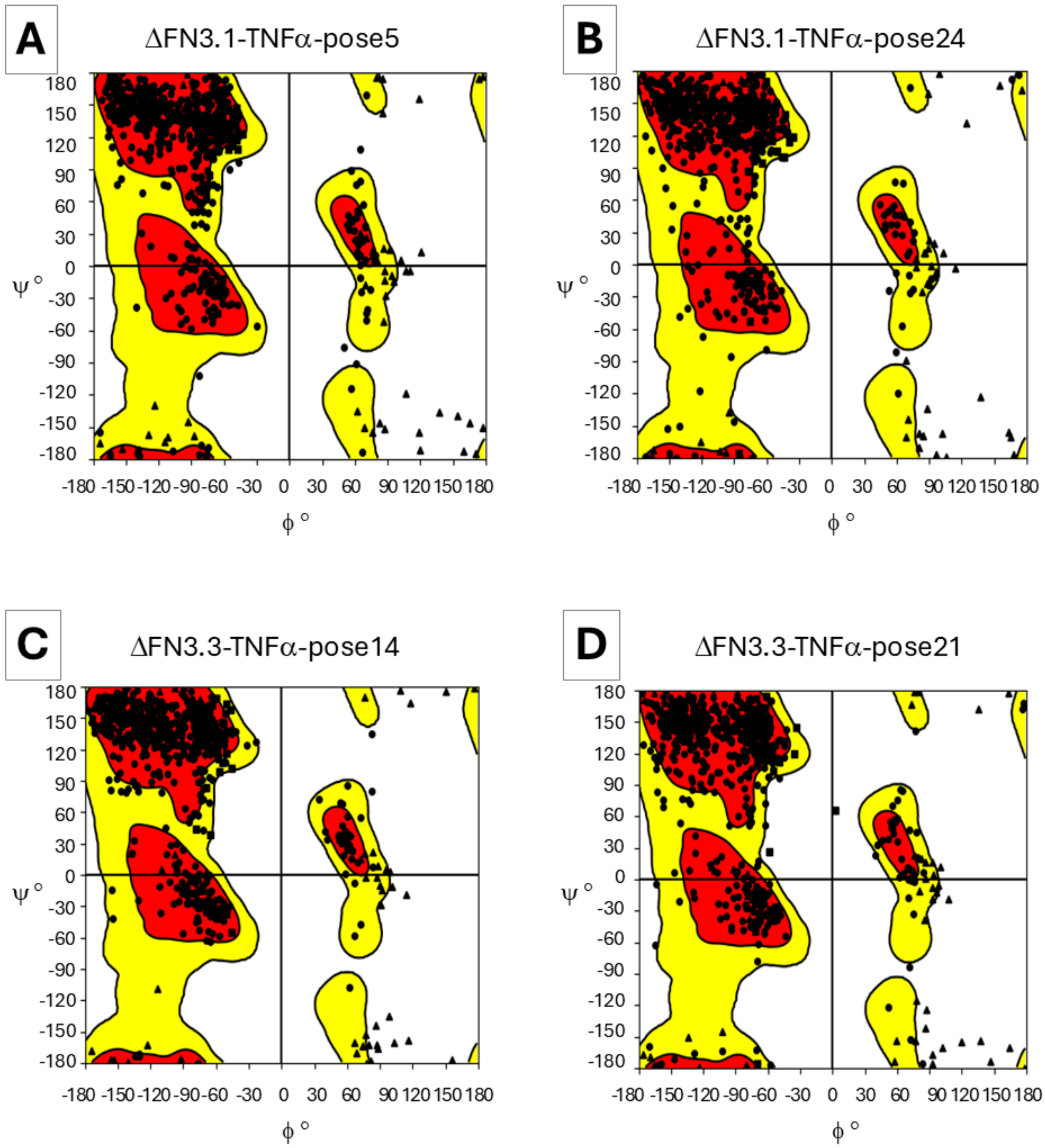

Figure S14. MD simulations of TNF-α-ΔFN3 complexes with different docking poses: (A) - ΔFN3.1- TNF-α-pose5; (B) - ΔFN3.1- TNF-α-pose24; (C) - ΔFN3.3- TNF-α-pose14; (D) - ΔFN3.3- TNF-α-pose21. Ramachandran map for representative MD simulation frames.

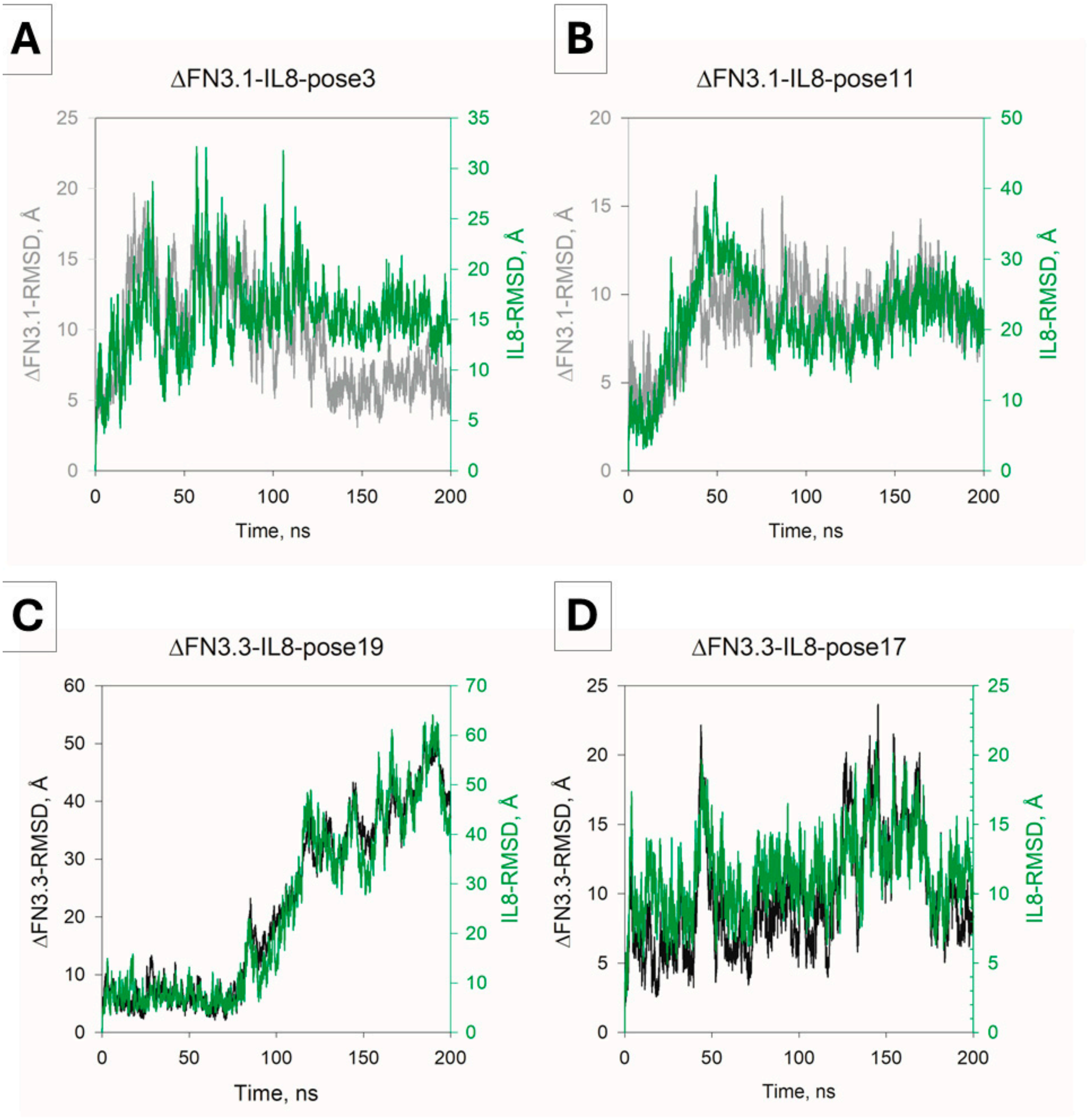

Figure S15. MD simulations of complexes IL8-ΔFN3: RMSD is used to measure average change in displacement of a selection of atoms for a particular frame with respect to a reference frame (A-D).

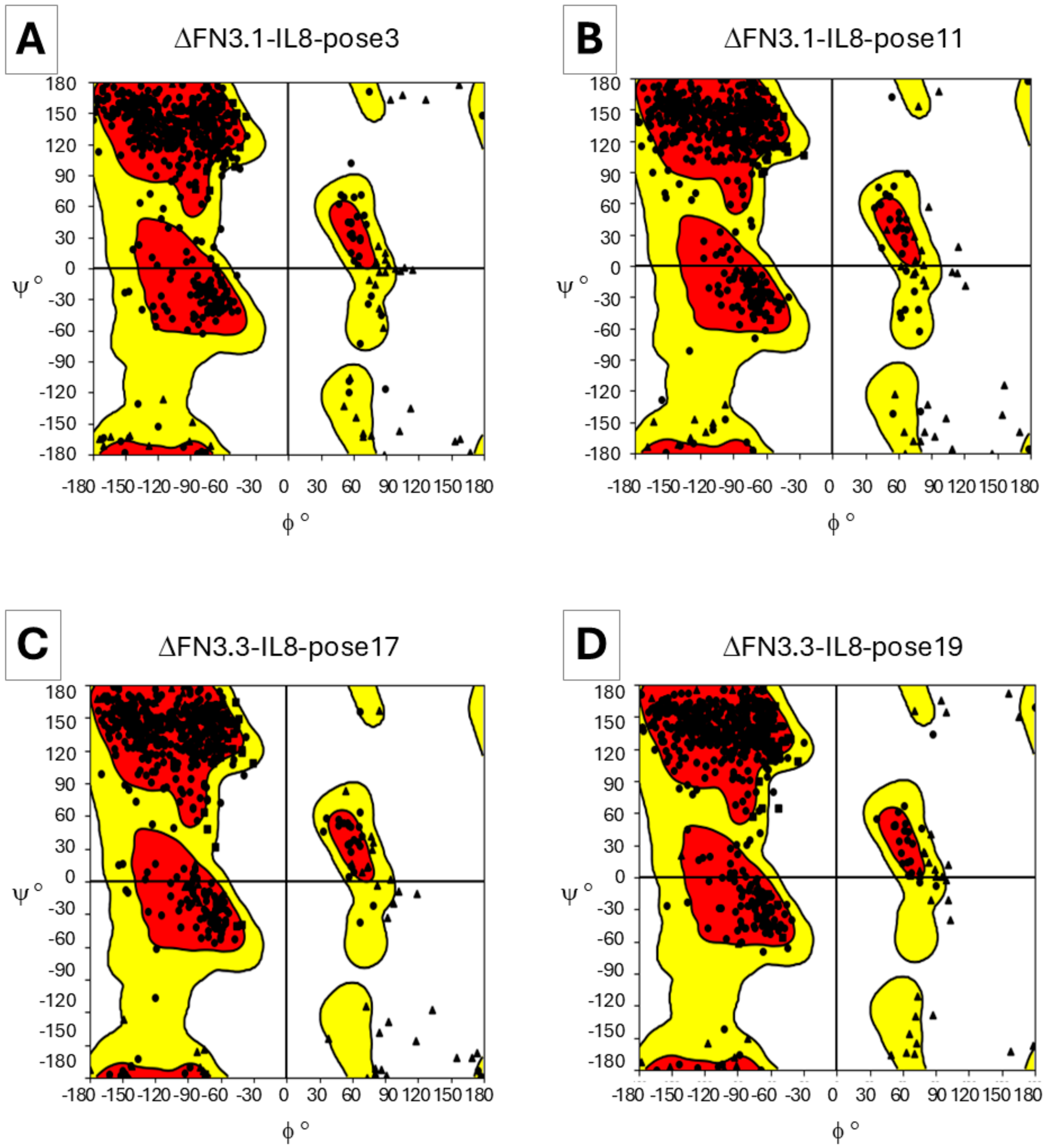

Figure S16. MD simulations of complexes IL8-ΔFN3: Ramachandran map for representative MD simulation frames (A-D).

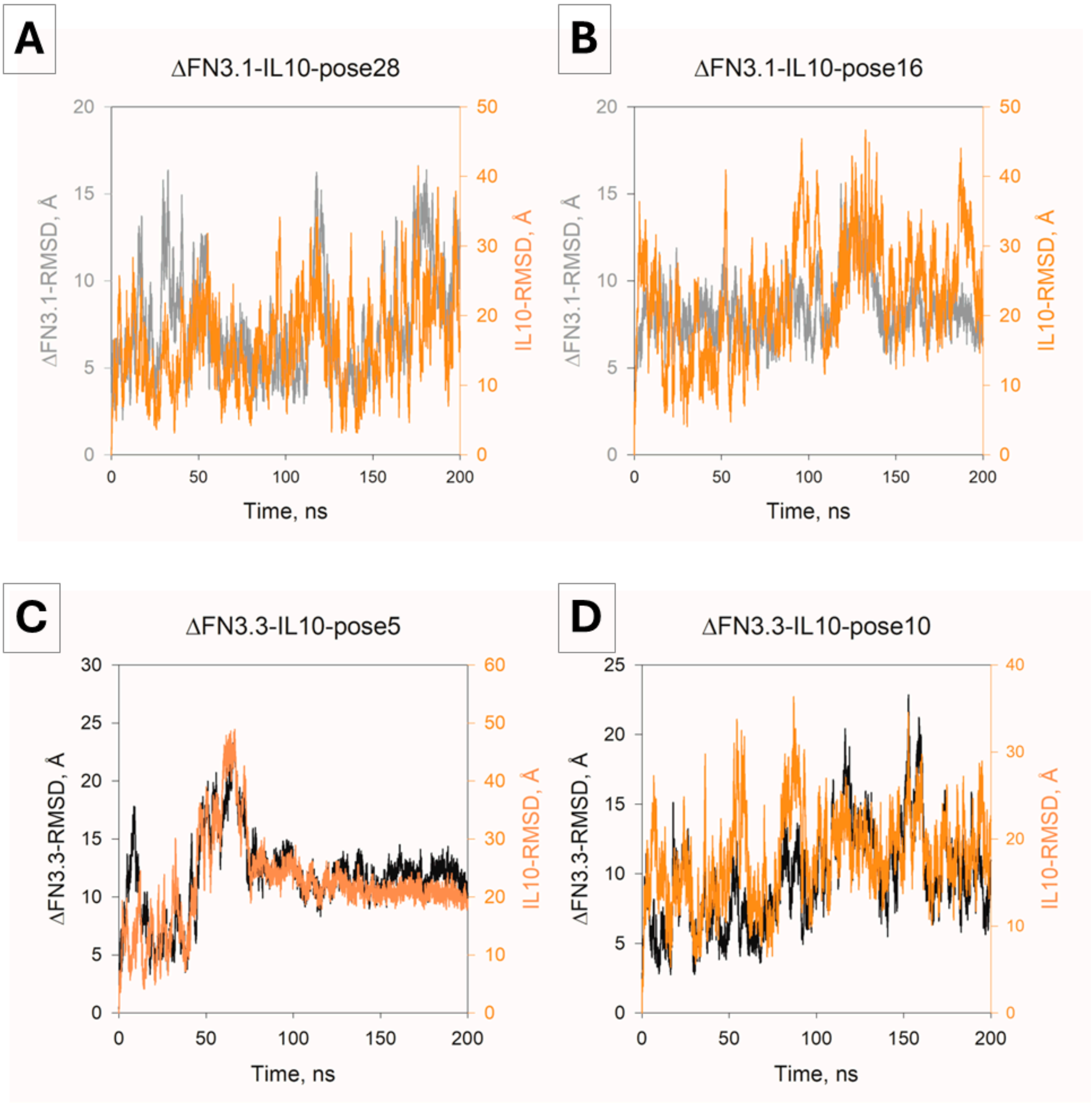

Figure S17. MD simulations of complexes IL10-ΔFN3: RMSD is used to measure average change in displacement of a selection of atoms for a particular frame with respect to a reference frame (A-D).

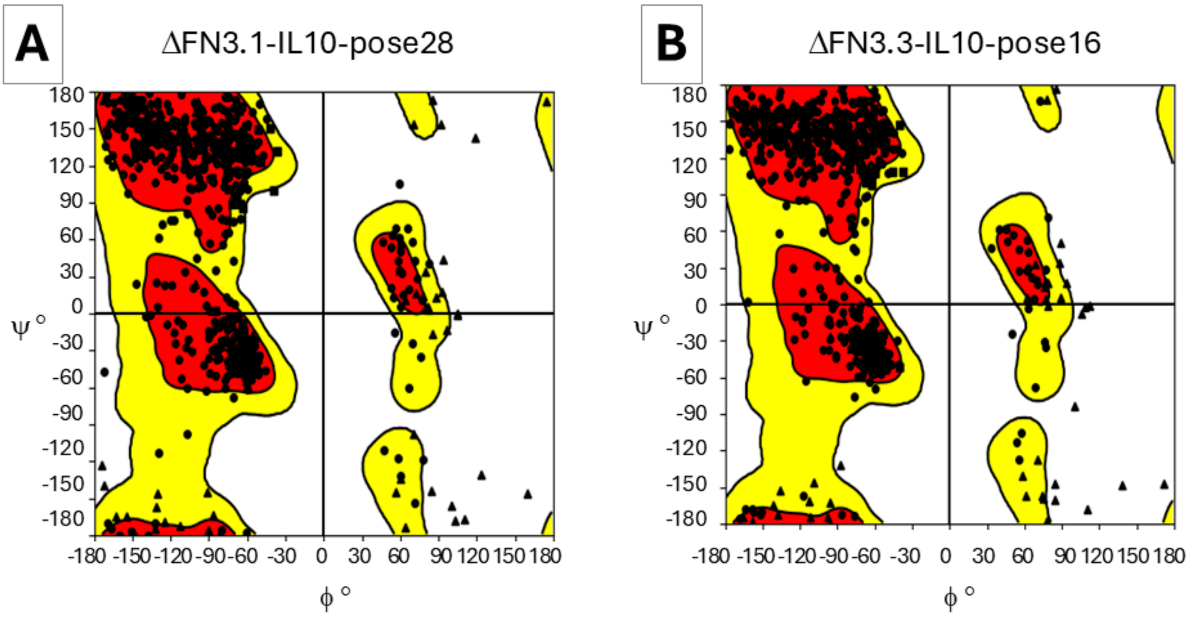

Figure S18. MD simulations of complexes IL10-ΔFN3.1: Ramachandran map for representative MD simulation frames (A,B).

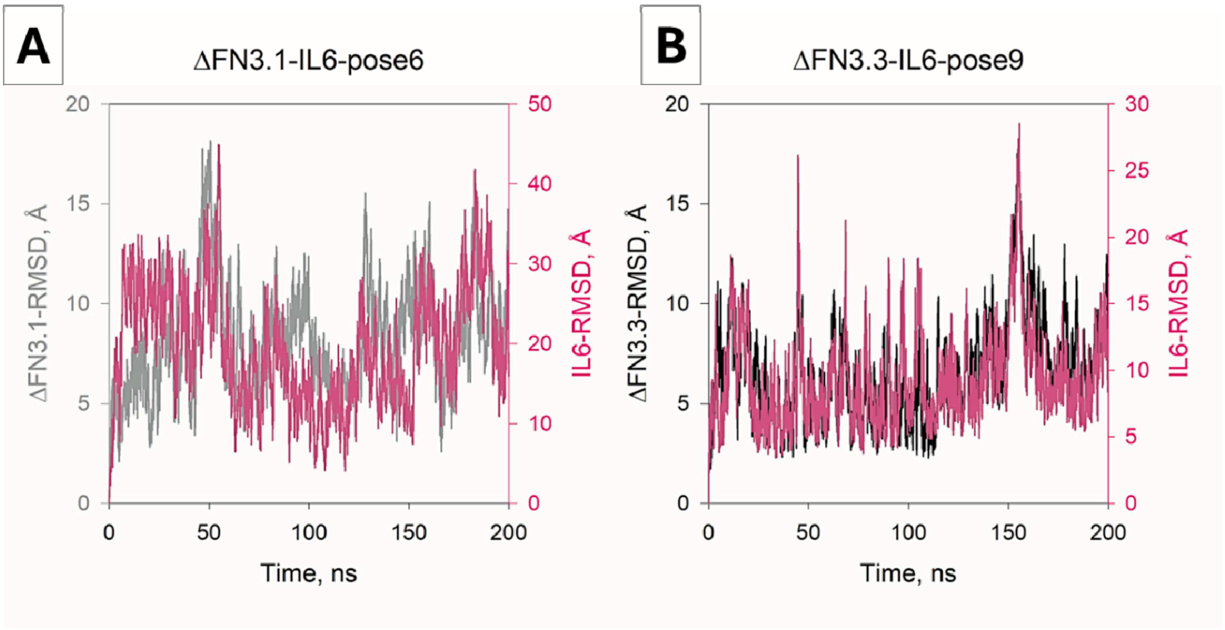

Figure S19. MD simulations of complexes IL6-ΔFN3: RMSD is used to measure average change in displacement of a selection of atoms for a particular frame with respect to a reference frame (A,B).

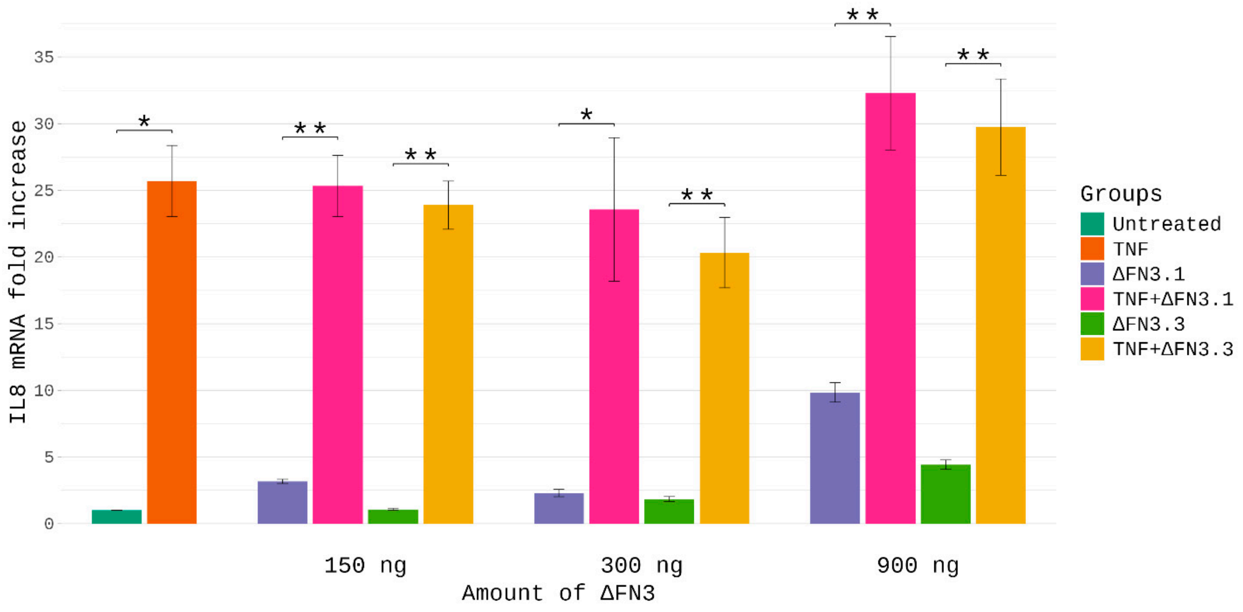

Figure S20. No additivity of combinations of ΔFN3 proteins and TNF-α on IL-8 mRNA. The mRNA levels in untreated cells were taken as 1. Asterisks, $p < 0.05$ (*), $p < 0.01$ (**) between the groups 'combination' vs. each protein alone.

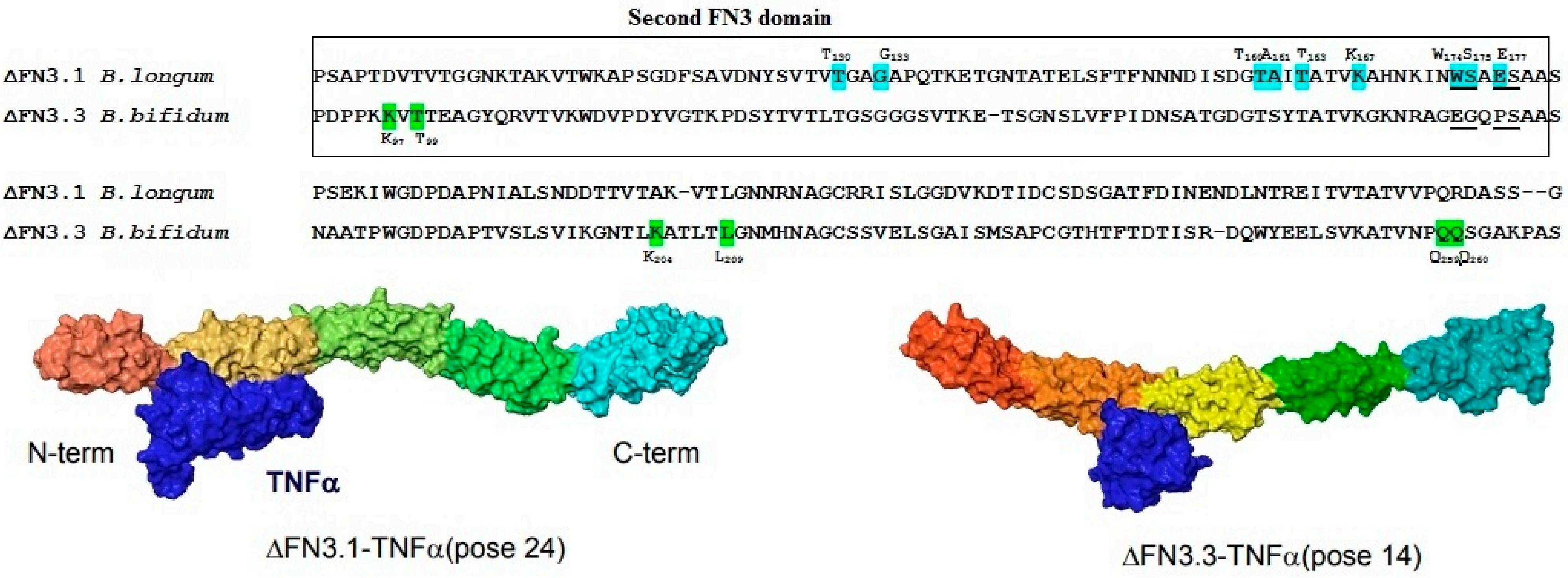

Figure 21. Interaction of TNF-α with surfaces of ΔFN3.1 *B. longum* and ΔFN3.3 *B. bifidum*: contact amino acids and optimal binding positions.

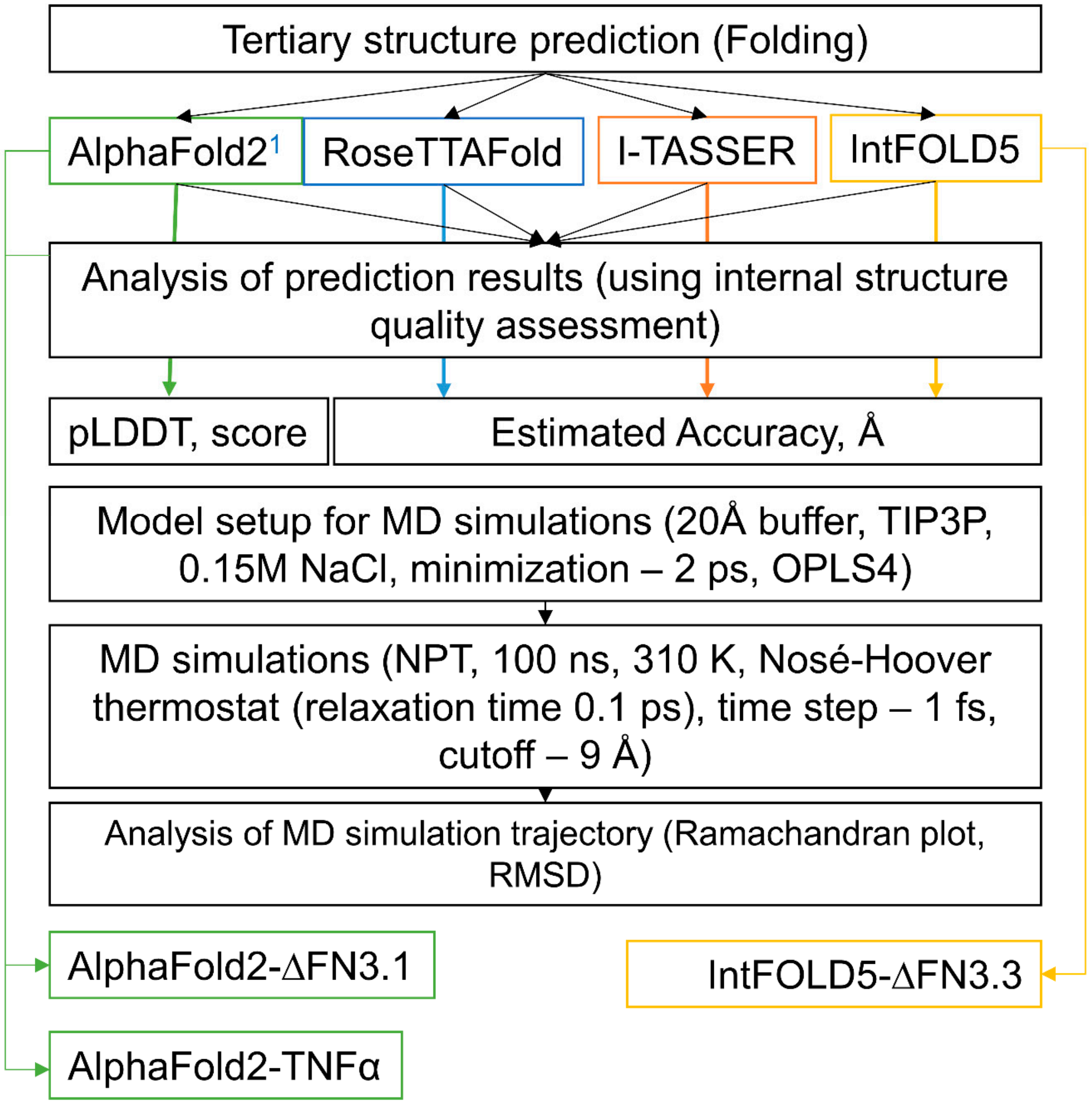

Figure S22. General algorithm for predicting tertiary structures.

Table S1. Folding results: pLDDT, predicted Local Distance Difference Test; C-score, Confidence Score; GMQS is Global Model Quality Score.

Application	Model	Scoring function	Structure
ΔFN3.1			
AlphaFold2	3	pLDDT = 91.3	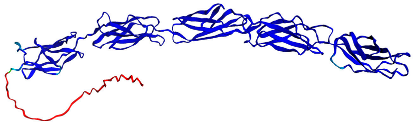
RoseTTAFold	1	pLDDT = 92.0	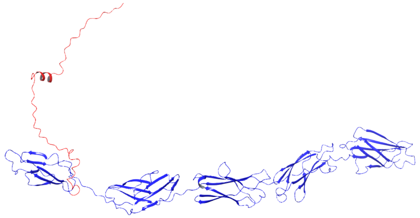
I-TASSER	1	C-score = -2.36	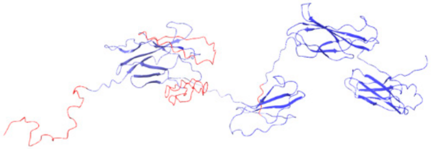
IntFOLD5	1	GMQS = 0.78	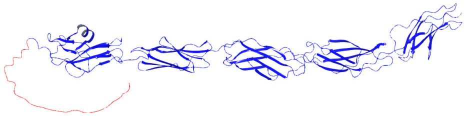
ΔFN3.3			
AlphaFold2	1	pLDDT = 90.4	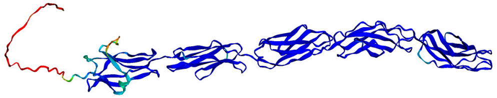
RoseTTAFold	1	pLDDT = 91.3	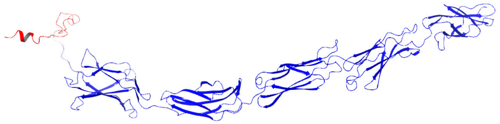

I-TASSER	1	C-score = -1.90	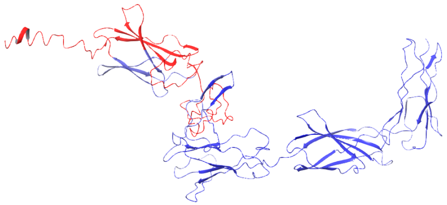
IntFOLD5	1	GMQS = 0.74	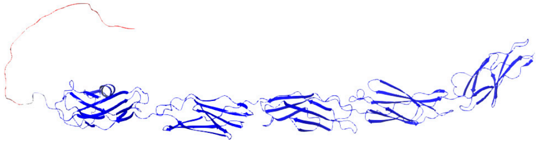
TNFα			
AlphaFold2	1	pLDDT = 93.7	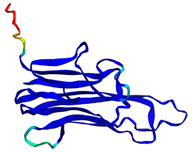
IL8			
AlphaFold2	2	pLDDT = 89.4	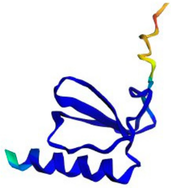
IL10			
AlphaFold2	2	pLDDT = 85.8	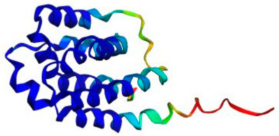
IL6			

AlphaFold2	2	pLDDT = 97.4	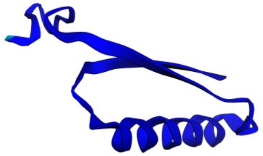

Table S2. Comparison of alignment score and RMSD of ΔFN3 structures obtained using different folding methodologies.

**Characteristic/protein**	**ΔFN3.1**		**ΔFN3.3**	
Methodology	AplhaFold2	IntFOLD5	AplhaFold2	IntFOLD5
Alignment score	0.374	0.387	0.563	0.265
RMSD, Å	3.056	3.102	3.710	2.460

Table S3. Docking of TNF-*α* to ΔFN3.1.

Pose	PIPER pose energy	PIPER pose score	Complex	Interaction			
				**ΔFN3.1**	**TNF-α**	**Distance, Å**	**Type**
5	-640.193	-247.137	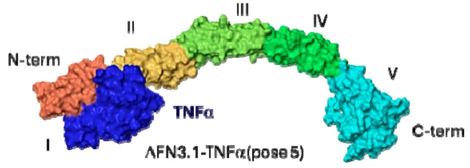	Thr143	Gln201	2.2	H-bond
				Asn124	Leu233	2.4	H-bond
				Val9	Thr155	1.6	clash
				Lys171	Val199	1.7	clash
				Pro24	Thr148	1.8	clash
20	-516.037	-138.705	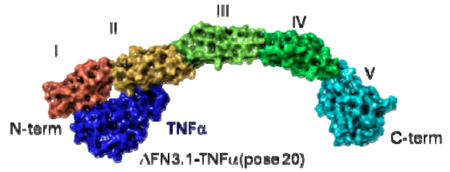	Trp174	His149	2.0	H-bond, π-π interaction
				Trp174	Gln178	1.6	clash
				Ile172	Ser175	2.1	H-bond
				Lys167	Glu180	1.9	H-bond
				Glu139	Gln178	2.4	H-bond
				Thr137	Arg179	1.8	H-bond, clash
24	-579.251	91.507	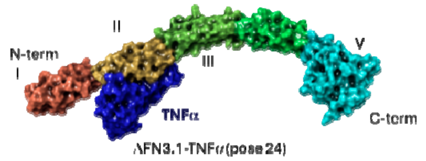	Trp 174	Gln 123	2.0	H-bond
				Asn 173	Arg 207	1.9	H-bond
				Gly 159	Ser 147	1.5	H-bond, clash
				Ser 157	Glu 180	2.3	H-bond
				Gly 133	His 149	1.8	H-bond
28	-519.188	9.206	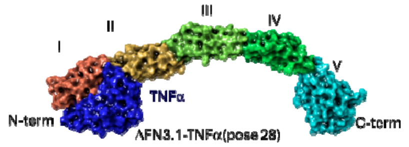	Ala 51	Thr 148	1.9	H-bond
				Pro 24	Arg 214	2.5	H-bond
				Thr 23	Leu 151	2.2	H-bond
30	-525.624	-101.848	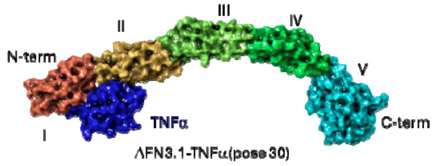	Trp 174	Ser 81	1.7	H-bond
				Lys 171	Tyr 195	1.6	H-bond

Table S4. Docking of TNF-*α* to ΔFN3.3.

Pose	PIPER pose energy	PIPER pose score	Complex	Interaction			
				ΔFN3.3	TNFα	Distance, Å	Type
14	-618.944	-157.185	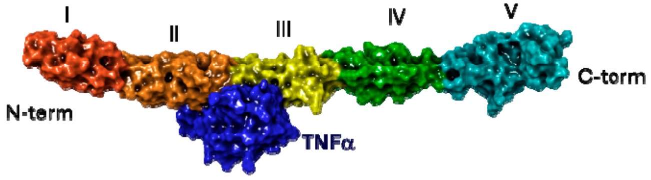 ΔFN3.3-TNFα(pose 14)	Gln 259	Tyr 195	2.4	H-bond
				His 235	Val 199	1.6	clash
				Gly 157	Asn 168	1.8	H-bond
				Thr 99	His 149	1.8	H-bond
				Lys 97	Glu 180	2.1	H-bond
21	-544.108	-47.546	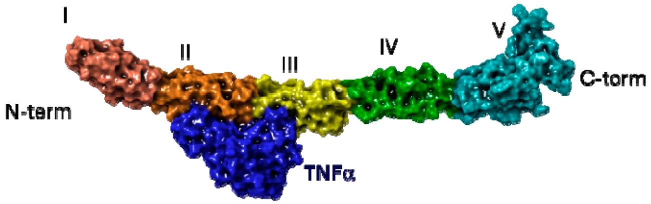 ΔFN3.3-TNFα(pose 21)	Gln 260	Leu 169	2.4	H-bond
				Gln 259	Asn 168	1.7	H-bond
				Gln 259	Thr 155	1.9	H-bond
				Asn 214	Ser 171	2.1	clash
				Ala 181	Gly 197	2.1	clash
				Lys 110	Gln 201	2.4	H-bond
				Thr 100	Val 199	2.1	H-bond
28	-599.140	-118.611	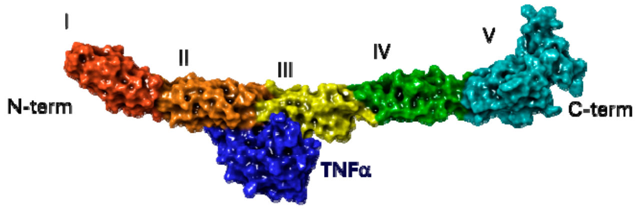 ΔFN3.3-TNFα(pose 28)	Gln 259	Leu 233	1.7	H-bond
				Asn 211	Tyr 195	2.3	H-bond
				Leu 209	Gly 197	2.2	H-bond
				Ala 181	Asp 86	2.1	H-bond
				Asn 180	Asp 86	2.1	H-bond
				Ser 159	Arg 82	2.3	H-bond
				Thr 158	Ser 81	1.4	clash
				Gly 157	Ser 81	1.8	clash
				Gly 157	Thr 83	1.7	H-bond

				Asp 156	Ser 81	2.0	clash
				Ser 152	Arg 82	2.3	H-bond
				Ser 131	Arg 82	1.7	clash

Table S5. MD trajectories of TNF-α in complexes with ΔFN3.

Complex	Pose	Intermolecular contacts in representative frames		Type of interactions	ΔG$_{bind}$, kcal/mol	K$_D$(calc), nM	K$_D$(exp), nM
		**ΔFN3**	**TNFα**				
ΔFN3.1- TNFα	24	A180	G100	H-bond	-116.6	2.14	**13.10±0.59**
		E180	Q101				
		W174	Q123				
		N173	Q123				
		H169	T165				
		T160	S147				
		G159	S147				
		D158	S147				
		D155	T148				
		S94	D121				
		K91	D121				
		D15	R207				
		D15	R120				
		D15	R207	Salt bridge			
	5	W174	Y195	π-π stacking	-106.3	**10.46**	
		H169	Y195				
		T150	R82	H-bond			
		N142	L233				
		G139	R78				
		K138	S80				
		T137	S80				
		S54	Q178				
		T21	S175				
		E12	Q178				

				Salt-bridge			
ΔFN3.3- TNFα	14	S261<br>Q260<br>Q260<br>Q259<br>C232<br>N214<br>D156<br>A102	Q178<br>S175<br>Q178<br>Y195<br>Y195<br>S171<br>S171<br>H149	H-bond	-96.98	**47.53**	**58.20±2.89**
				Salt bridge			
	21	N211<br>T208<br>K204<br>T192<br>A190<br>S176<br>P175<br>T161<br>D156	N168<br>R158<br>E129<br>T163<br>T163<br>R179<br>R179<br>Q178<br>N168	H-bond	-78.11	1017.18	
		K204<br>K165	E129<br>E180	Salt bridge			

Table S6. Docking of interleukins to ΔFN3.1.

Complex	PIPER pose energy	PIPER pose score	Complex	Interaction			
				ΔFN3.1	IL	Distance, Å	Type
ΔFN3.1-IL8(pose3)	-671.541	-50.737	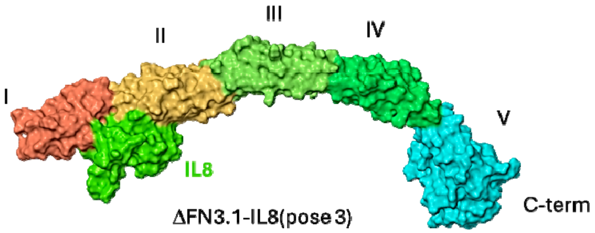	Asn173	Glu53	1.6	H-bond
				Pro13	Arg52	2.0	H-bond
				Glu12	Arg 2	2.6	salt bridge
				Thr21	Lys25	2.2	H-bond
				Glu86	Lys20	1.9	H-bond salt bridge
				Asp90	Tyr18	2.1	H-bond
				Asp123	Lys16	1.9	H-bond
				Glu139	Arg11	1.8	H-bond
				Thr137	Arg11	2.0	H-bond
				Lys167	Glu9	2.0	salt bridge
ΔFN3.1-IL8(pose11)	-536.911	-36.931	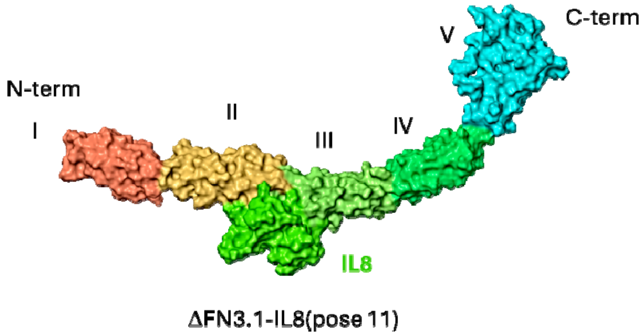	Lys185	Pro21	1.9	H-bond
				Gly159	Lys20	1.9	H-bond
				Asp158	Tyr18	1.9	H-bond
				Gly217	Tyr18	2.0	H-bond
				Cys218	Lys16	2.2	H-bond
				Gln261	Lys16	2.2	H-bond
				Arg220	Cys12	2.0	H-bond
				Asp232	Arg11	2.2	H-bond
				Asp235	Arg11	2.3	H-bond
				Arg220	Glu9	1.9	H-bond salt bridge
				Arg219	Glu9	2.2	H-bond
				Asp419	Asn160	2.2	H-bond
				Asp419	Lys157	3.1	salt bridge

<table>
<tr><td rowspan="6">ΔFN3.1-IL10(pose16)</td><td rowspan="6">-591.337</td><td rowspan="6">89.640</td><td rowspan="6">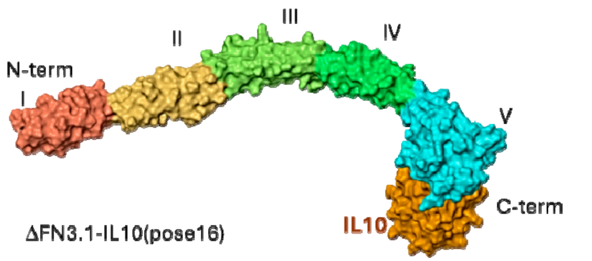
</td><td>Asp422</td><td>Arg110</td><td>1.9</td><td>H-bond</td></tr>
<tr><td>Thr497</td><td>Asn18</td><td>1.7</td><td>H-bond</td></tr>
<tr><td>His499</td><td>His14</td><td>1.6</td><td>H-bond</td></tr>
<tr><td>Gly439</td><td>Ser1</td><td>2.1</td><td>H-bond</td></tr>
<tr><td>Glu12</td><td>Arg110</td><td>2.0</td><td>H-bond, salt bridge</td></tr>
<tr><td>Thr21</td><td>His14</td><td>2.0</td><td>H-bond</td></tr>
<tr><td rowspan="3">ΔFN3.1-IL10(pose28)</td><td rowspan="3">-521.380</td><td rowspan="3">-53.366</td><td rowspan="3">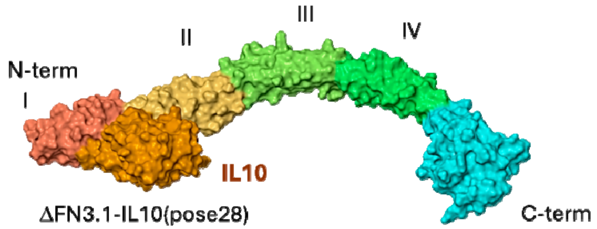
</td><td>Glu139</td><td>Lys57</td><td>1.9</td><td>H-bond, salt bridge</td></tr>
<tr><td>Lys171</td><td>Lys157</td><td>2.1</td><td>H-bond</td></tr>
<tr><td>Asp422</td><td>Lys132</td><td>2.1</td><td>H-bond, salt bridge</td></tr>
<tr><td rowspan="4">ΔFN3.1-IL6(pose6)</td><td rowspan="4">-597.360</td><td rowspan="4">12.487</td><td rowspan="4">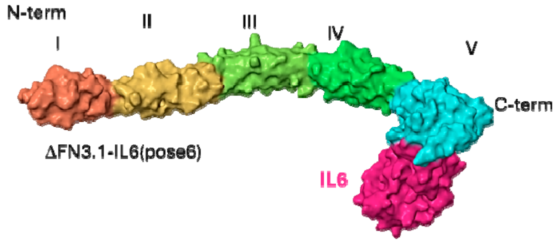
</td><td>Phe410</td><td>His16</td><td>2.6</td><td>π-π - interaction</td></tr>
<tr><td>Asp422</td><td>His16</td><td>1.9</td><td>H-bond</td></tr>
<tr><td>Asn413</td><td>Pro15</td><td>2.4</td><td>H-bond</td></tr>
<tr><td>Lys481</td><td>Asp8</td><td>2.0</td><td>H-bond, salt bridge</td></tr>
</table>

Table S7. Docking of interleukins to ΔFN3.3.

<table>
<tr><th rowspan="2">Complex</th><th rowspan="2">PIPER pose energy</th><th rowspan="2">PIPER pose score</th><th rowspan="2">Complex</th><th colspan="4">Interaction</th></tr>
<tr><th>ΔFN3.3</th><th>IL</th><th>Distance, Å</th><th>Type</th></tr>
<tr><td rowspan="10">ΔFN3.3-IL8(pose17)</td><td rowspan="10">-604.767</td><td rowspan="10">183.363</td><td rowspan="10">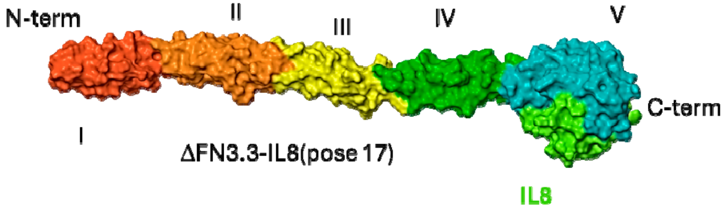
</td><td>Asn501</td><td>Glu 9</td><td>1.6</td><td>H-bond</td></tr>
<tr><td>Asn501</td><td>Gln13</td><td>1.7</td><td>H-bond</td></tr>
<tr><td>Ser 500</td><td>Glu 9</td><td>1.8</td><td>H-bond</td></tr>
<tr><td>Phe489</td><td>Glu53</td><td>1.8</td><td>H-bond</td></tr>
<tr><td>Asp436</td><td>Lys 8</td><td>2.1</td><td>H-bond, salt bridge</td></tr>
<tr><td>Asp429</td><td>Lys47</td><td>2.2</td><td>H-bond</td></tr>
<tr><td>Gln 425</td><td>Lys28</td><td>2.1</td><td>H-bond</td></tr>
<tr><td>Thr 418</td><td>Lys28</td><td>1.7</td><td>H-bond</td></tr>
<tr><td>Asp414</td><td>Arg5</td><td>1.9</td><td>H-bond, salt bridge</td></tr>
<tr><td>Glu 412</td><td>Ala 1</td><td>1.9</td><td>H-bond, salt bridge</td></tr>
<tr><td rowspan="5">ΔFN3.3-IL8(pose19)</td><td rowspan="5">-538.032</td><td rowspan="5">-79.599</td><td rowspan="5">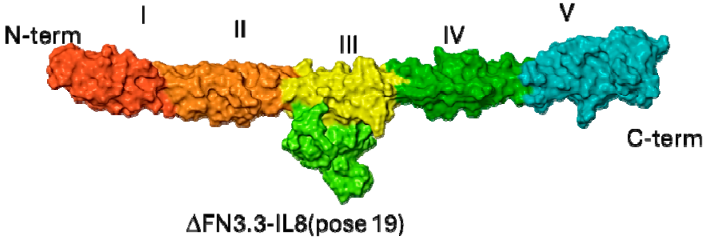
</td><td>Leu209</td><td>Glu9</td><td>1.9</td><td>H-bond</td></tr>
<tr><td>His235</td><td>Glu9</td><td>2.2</td><td>H-bond</td></tr>
<tr><td>Asp156</td><td>Arg11</td><td>2.0</td><td>H-bond, salt bridge</td></tr>
<tr><td>Gly210</td><td>Arg11</td><td>1.8</td><td>H-bond</td></tr>
<tr><td>Thr238</td><td>Gly51</td><td>2.1</td><td>H-bond</td></tr>
<tr><td rowspan="11">ΔFN3.3-IL10(pose5)</td><td rowspan="11">-637.170</td><td rowspan="11">94.313</td><td rowspan="11">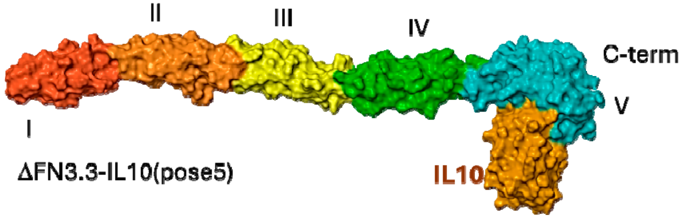
</td><td>Ser491</td><td>Arg106</td><td>2.1</td><td>H-bond</td></tr>
<tr><td>Ser491</td><td>Ser66</td><td>1.8</td><td>H-bond</td></tr>
<tr><td>Ser491</td><td>Gln63</td><td>1.6</td><td>H-bond</td></tr>
<tr><td>Arg487</td><td>Gln63</td><td>2.0</td><td>H-bond</td></tr>
<tr><td>Arg487</td><td>Asn116</td><td>2.1</td><td>H-bond</td></tr>
<tr><td>Glu477</td><td>Lys117</td><td>1.9</td><td>H-bond</td></tr>
<tr><td>Arg434</td><td>Glu9</td><td>1.9</td><td>H-bond</td></tr>
<tr><td>Ser432</td><td>His109</td><td>2.2</td><td>H-bond</td></tr>
<tr><td>Gln425</td><td>Asn116</td><td>1.8</td><td>H-bond</td></tr>
<tr><td>Asp414</td><td>Arg110</td><td>2.0</td><td>H-bond</td></tr>
<tr><td>Ser413</td><td>Asn160</td><td>2.0</td><td>H-bond</td></tr>
</table>

				Thr397	Arg159	2.1	H-bond
ΔFN3.3-IL10(pose10)	-548.008	-18.985	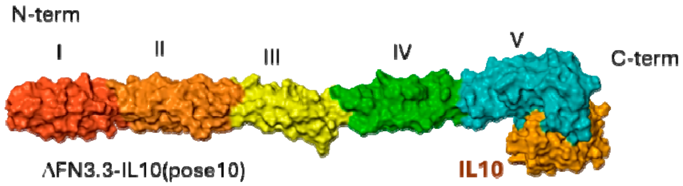	Asp414	Arg102	2.1	H-bond, salt bridge
				Glu412	Arg107	2.0	H-bond
				Glu412	Gln7	2.0	H-bond
				Ser500	Tyr59	2.1	H-bond
				Thr405	Arg102	2.4	H-bond
				Asp414	Arg106	2.1	H-bond
				Asp414	Arg107	2.0	H-bond
				Ser413	Arg107	1.8	H-bond
				Asn501	His109	2.1	H-bond
				Asn501	Cys114	2.3	H-bond
				Asn501	Glu115	2.1	H-bond
				Asp436	Lys117	2.0	H-bond
ΔFN3.3-IL6(pose9)	-632.637	97.885	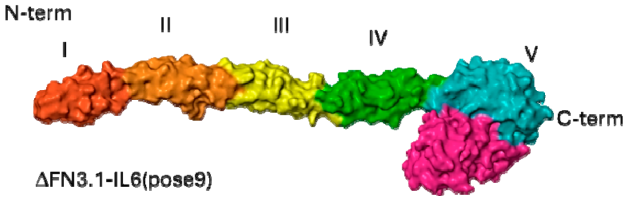	Arg502	Asp11	2.0	H-bond, salt bridge
				Thr497	Asp11	2.5	H-bond
				Pro493	Lys10	1.5	clash
				Ser491	Lys10	1.8	H-bond
				Gly490	Arg17	1.8	H-bond
				Asn416	Arg25	2.3	H-bond

Table S8. MD trajectories of interleukins in complexes with ΔFN3.

Complex	Pose	Intermolecular contacts formed in representative frames		Type of interactions	ΔG$_{bind}$, kcal/mol	K$_D$(calc), nM	K$_D$(exp), nM
		ΔFN3	IL				
ΔFN3.1- IL8	3	N173	E53	H-bond	-109.49	6.20	**4.94±0.21**
		P13	R52				
		D90	R52				
		Q14	D50				
		D15	D50				
		A10	K20				
		G11	Y18				
		P8	K16				
		I172	Q13				
		W174	Q13				
		E139	R11				
		T137	K8				
		E139	S6				
		S126	S6				
		D90	R52	Salt bridge			
	11	D263	D50	H-bond	-60.29	18354.81	
		D263	K28				
		R262	D50				
		D229	K8				
		R219	E53				
		D263	K28	Salt-bridge			
		D263	K47				

		R262<br>D229<br>R219	D50<br>K8<br>E53				
ΔFN3.3- IL8	17	R502<br>N501<br>N501<br>N501<br>G490<br>S488<br>T447<br>R434<br>F433<br>S432<br>S432<br>D414<br>E412<br>T410<br>T405<br>S403	Q13<br>K8<br>E9<br>R11<br>E53<br>E53<br>R73<br>E43<br>Q13<br>Q13<br>R31<br>R5<br>A1<br>R5<br>R5<br>R5	H-bond	-119.80	**1.17**	**2.25±0.11**
		D436<br>D414<br>E412	K8<br>R5<br>A1	Salt-bridge			
	19	T236<br>H235<br>G233<br>G233<br>S216	E53<br>E53<br>Q13<br>R31<br>K16	H-bond	-80.12	733.98	

		D156	R11				
		D156	R11	Salt bridge			
ΔFN3.1- IL10	28	W174	K57	H-bond	-100.61	26.39	**62.2±3.09**
		N173	Y59				
		E139	K57				
		D123	R159				
		S19	N10				
		S19	T13				
		Q14	E9				
		Q14	N10				
		E12	K157				
		G11	N160				
		V9	N160				
		E139	K57	Salt bridge			
		D123	R159				
		E12	K157				
	16	Q501	R24	H-bond	-99.21	33.09	
		S495	T155				
		G489	R159				
		A480	R159				
		K471	N160				
		D422	S1				
		D422	G3				
		Q416	I158				
		T411	Q4				
		D475	K57	Salt bridge			

Table S9. Description of experimental groups. Untreated THP-1 cells were taken as 1.

Number	Name	TNF-α, ng	ΔFN3.1, ng	ΔFN3.1, ng
1	Untreated	-	-	-
2	TNF	300	-	-
3	FN3.1	-	300	-
4	FN3.3	-	-	300
5	TNF+FN3.1	300	300	-
6	TNF+FN3.3	300	-	300
7	FN3.1_0.5	-	150	-
8	FN3.3_0.5	-	-	150
9	FN3.1_3	-	900	-
10	FN3.3_3	-	-	900
11	TNF+FN3.1_0.5	300	150	-
12	TNF+FN3.3_0.5	300	-	150
13	TNF+FN3.1_3	300	900	-
14	TNF+FN3.3_3	300	-	900

Table S10. Primers used in RT-PCR.

**Target gene**	**Direction**	**Nucleotide sequence (5′→3′)**
HPRT1	Forward	TATATCCAACACTTCGTGGGGTC
	Reverse	ACAGGACTGAACGTCTTGCT
TNFa	Forward	TCTCCTTCCTGATCGTGGCA
	Reverse	TATCTCTCAGCTCCACGCCA
IL8	Forward	TGGCTCTCTTGGCAGCCTTC
	Reverse	TGCACCCAGTTTTCCTTGGG
IL6	Forward	ACAGCCACTCACCTCTTCAG
	Reverse	TGGGTCAGGGGTGGTTATTG
